# Students as stakeholders in assessment: how students perceive the value of an assessment

**DOI:** 10.1007/s40037-018-0480-3

**Published:** 2018-11-12

**Authors:** Michelle Ricci, Christina St-Onge, Jing Xiao, Meredith Young

**Affiliations:** 1 Dalhousie University, Halifax, Nova Scotia Canada; 2 Department of Medicine and Health Sciences, Université de Sherbrooke, Sherbrooke, Canada; 3 Centre for Medical Education, McGill University, Montreal, Canada

**Keywords:** Assessment, Stakeholder Acceptability, Validity, Feedback, Multiple-Choice Questions

## Abstract

**Introduction:**

For assessment to fill an educational role, students must see the results generated by assessment as valuable, and actively engage with this feedback in order to support learning. Few studies include examinees as stakeholders in validation beyond general notions of acceptability. Here, we explore students as stakeholders in the validation of a newly implemented assessment.

**Methods:**

A student-relevant validity framework based on the unified theory of validity was created and adapted to a survey format. Likert-style items were used to examine first- and second-year medical students’ perceptions of a new cumulative assessment, with several open-ended items. Analysis included: mean ratings per subscale of validity evidence, thematic analysis of comments, and a correlation between questionnaire subscores and exam performance.

**Results:**

Seventy-seven students participated (20.5%). Student perceptions of the assessment were favourable, with significantly different ratings across validity evidence (Response Process (4.8 (*SD* = 0.7); scored/6), Content (4.6(0.9)), Consequential (4.4(0.8)), Internal Structure (4.2(0.9)), and Relationship to Other Variables (4.0(1.0))). Exam performance correlated with subscores for Relationship to Other Variables (*r* = 0.34, *p* < 0.005) and Response Process (*r* = 0.24, *p* < 0.05).

**Discussion:**

Students perceived the assessment as facilitating learning, providing ‘checkpoints’, and were disappointed when it did not meet their expectations regarding the purpose of assessment. If students perceive that results do not reflect their future performance in clinical environments, or do not align with their perceived purpose of assessment, the educational value of assessment may be limited. It is critical to understand when, and how students engage in interpreting and integrating assessment-generated feedback to ensure that assessment contributes positively to learning.

## What this paper adds

In order for assessment to support future learning, students must engage with the results of assessment to identify areas for future learning. We adapt a validity framework to be student-relevant, and apply this framework to evaluate a newly implemented assessment. Students rated the exam well overall, commented on the educational value of the assessment, but were disappointed when they felt that the educational goals of assessment were not being met. This study suggests that students are able to engage critically with assessment, and lays a foundation for considering assessees as stakeholders beyond simplistic notions of acceptability.

## Introduction

Assessment is a key component in the education of healthcare professionals. It has several education roles [1–5] such as providing direction and motivation for further learning [6–8], supporting future learning [6, 8–12], and identifying areas for improvement [6, 12]. For assessments to fulfil these roles defensibly, it must be of high quality. Several criteria exist for ‘good assessment’ including: validity, coherence, reproducibility-consistency, equivalence, feasibility, educational effect, catalytic effect, and acceptability [13, 14]. The relative importance of these criteria varies depending on the type of assessment (formative vs. summative), the stakes of the assessment (high vs. low), and the consideration for stakeholders’ needs. A stakeholder can be any individual who has an interest in the outcome of the assessment (e. g. Norcini & McKinley [15]), including the general public, faculty/teachers, health system/regulators, and examinees themselves. Exploring the perceived value of an assessment tool, including perceptions of acceptability, credibility, and fairness could be one means to include different stakeholders in the assessment process (e. g. Norcini et al. [14]; Van Der Vleuten [16]).

If we focus on the presumed educational roles of assessment, it is not unreasonable to consider students as relevant stakeholders in the context of assessment for learning [6, 8–12, 17–19]. Students must be able to receive, interpret, and infer meaning behind their assessment score in order for assessment to be used to provide direction and motivation for learning [6–12] and to identify areas for improvement [6, 20]. Students report that formative assessment can enhance the learning process, provided that they perceive the assessment to be effective [17]. Trainees who perceive an assessment process as ‘unfair’ (i. e. ineffective) may be less likely to accept the feedback provided [21, 22], and more likely to dismiss important feedback when they consider the assessment process to be flawed [21–23]. This active dismissal of assessment-generated feedback likely results in missed learning opportunities, which in turn undermines the educational role of assessment. Therefore, student perceptions of the value of an assessment can have downstream effects beyond whether students ‘like’ a particular assessment tool or approach [14, 17, 18].

We suggest that in order to facilitate the integration of assessment feedback to support learning, stakeholder perceptions should move away from simplistic notions of face-value acceptability (as whether or not a particular stakeholder group ‘likes it’) into broader notions of how stakeholders perceive the value of assessments. In order to investigate students’ perceptions of the value of a newly implemented assessment, the goals of this study were twofold: (1) to adapt a validity framework to be used to document students’ perceived value of assessment, contextualized according to an adaptation of Messick’s validity evidence [24]; (2) use the student-relevant validity framework to explore how students perceive the value of a newly implemented assessment.

## Methods

### Participants

#### *Recruitment*:

Students from the undergraduate medical class of 2017 (Year 2; *n* = 185) and class of 2018 (Year 1; *n* = 191) from a Canadian University were invited to complete a questionnaire by email. Each class was sent an email from their respective course administrator 1 day after completing a newly implemented multiple-choice cumulative exam. Another email was sent 1 day after receiving the exam results. For each round of data collection, the survey remained open for 3 days and no reminders were sent due to the presence of multiple rounds of data collection and limitations put in place to protect participant identity.

#### *Incentive:*

Students who chose to participate in this study could submit their email address for a draw for one of five $25 gift cards. Five gift cards were available per cohort (Year 1 and Year 2) for each questionnaire period (post examination and post release of the examination results).

### Questionnaire development

Within an assessment for learning perspective, students are expected to receive their assessment results, interpret them in the context of their current knowledge and performance, and identify areas for improvement. This necessitates that students interpret their assessment scores in order to shape further learning. Therefore, to explore students’ perceived value of assessment, we chose to adopt five categories of validity evidence in Messick’s unified theory of validity [24] as a base for our questionnaire.

Several works have translated Messick’s framework [24] into actionable targets for the collection of validity evidence (i. e. [25–27]), and as such, we believed it was the best framework to operationalize into statements applicable to students’ interactions with assessment (including items related to: content, response process, internal structure, relationship to other variables, and consequential). Further, we believed that the unified theory of validity [24], as translated for the health professions education (HPE) context [27], provided a structured approach to identifying potential areas of student-relevant validity evidence, rather than relying on a more inference-based validity framework (e. g. Kane, [28]) given the exploratory nature of this study.

In order to adapt an existing validity framework to align with a student-as-score-interpreter lens, we relied on DeVellis’ [29] 8-step approach to tool development and validation to create a questionnaire to examine student perceptions of the value of assessment. We focused on development Steps 1–4, and details regarding each step of tool development will be reported here.

Step I: We created a student-relevant definition for each of the five categories of validity evidence in the unified theory of validity [24]. We relied on the definitions of each category of evidence translated to a health professions context [27], and translated them to be relevant to a student perspective (Tab. 1).

**Table 1:** Type of validity evidence, and how each type of validity evidence was translated into a student-relevant definition, and individual survey items

Evidence of validity	Definition as per Cook & Beckman 2006 [27]	Proposed student relevant definition	Individual items
Content	*Comprises a description of steps taken to ensure that assessment content (including scenarios, questions, response options, and instructions) reflects the construct it is intended to measure (e.* *g., ‘professionalism’). This might involve basing the assessment on prior instruments, obtaining expert review, or using an assessment blueprint*	*Students feel the exam meets their expectations in terms of content, level of difficulty, breadth of topics covered, and alignment with curricular objectives*	1. The breadth of material covered on the R&E cumulative exam was appropriate
			2. The content of the R&E cumulative exam reflects the learning objectives of the R&E week
			3. The R&E cumulative exam was at the level of difficulty that I expected
			4. The R&E cumulative exam questions were appropriately weighted across all blocks
			5. The R&E cumulative exam was fair
Response process	*Comprises theoretical and empirical analyses evaluating how well rater or examinee actions (responses) align with the intended construct. This includes assessment security (those who cheat are not responding based on the intended construct), quality control, and analysis of examinees’ or raters’ thoughts or actions during the assessment activity*	*Students feel the exam administration and scoring process is fair, and that there are appropriate quality control measures in place (e.* *g., monitoring, consequences for cheating behaviour) that allow for an appropriate assessment of their mastery of the material*	6. The R&E cumulative exam invigilation was effectively performed during the exam
			7. The R&E cumulative exam was administered in a way that allows true reflection of individual student mastery of the required material
			8. If a student were to act dishonestly during the R&E cumulative exam (e. g., cheating) they would be caught
			9. There is an appropriate process in place to address students who behave dishonestly (e. g., cheating)
Internal structure	*Comprises data evaluating the relations among individual assessment items and how these relate to the overarching construct. This most often takes the form of measures of reproducibility (reliability) across items, stations, or raters, but can also include item analysis (item difficulty and item discrimination) and factor analysis*	*Students feel that the range of item difficulty and discrimination is appropriate and that the exam is reliable; therefore, they are comfortable with the interpretation of the scores*	10. The questions on the R&E cumulative exam allow for differentiating between students who master the content and students who do not
			11. The range of difficulty of questions on the R&E cumulative exam appropriately reflects the diversity of experiences encountered in a clinical setting
			12. The R&E cumulative exam results are a fair portrayal of what I believe my level of clinical knowledge to be
			13. My performance on R&E cumulative exams is consistent across exams
Relationship to other variable	*Regards the statistical associations between assessment scores and another measure or feature that has a specified theoretical relationship. This relationship might be strongly positive (e.* *g., two measures that should measure the same construct) or negligible (for measures that should be independent)*	*Students feel that the exam is aligned with clinical scenarios, and builds on the foundation set by the block exams*	14. My performance on the R&E cumulative exam reflects my performance in a clinical setting
			15. My R&E cumulative exam scores are a more appropriate representation of my level of mastery than my block exam scores
			16. The block exams provide me with adequate foundational knowledge to succeed in the R&E cumulative exam
			17. There is a disconnect in my performance on the block exams the R&E cumulative exam^a^
			18. My performance on the R&E cumulative exam gives me confidence in my ability to perform in the clinical setting
Consequential	*Regards the impact, beneficial or harmful, of the assessment itself and the decisions and actions that result (e.* *g., remediation following sub-standard performance). This also includes factors that directly influence the rigor of such decisions, such as the definition of the passing score (e.* *g., at what point is remediation required?) and differences in scores among subgroups where performance ought to be similar (suggesting that decisions may be spurious)*	*Students perceive the exam as having more positive consequences (e.* *g., promotes learning and reflection) than negative consequences (e.* *g., failing a student that has mastered the content), and that there is a consideration for social consequences*	19. The R&E cumulative exam helps to prepare me for work in a clinical setting
			20. The R&E cumulative exams are appropriate checkpoints before entering a clinical setting
			21. The R&E cumulative exam causes me more anxiety than the block final exam^a^
			22. The students who fail the R&E week exams are those who did not master the exam content
			23. The learning experience of the R&E week exam is an overall positive learning experience
			24. The procedures in place for a failed R&E cumulative exam will be beneficial to my development

*Note: R&E* refers to Reflection and Evaluation cumulative exams

^a^Indicates items that were reverse coded

Step II and III: Up to 10 items were generated by the research team per category of validity evidence. Iterative review and refinement of the items was done by three team members with varying backgrounds and expertise; MR: an undergraduate medical student at the time of the study, MY: expertise in assessment and the local context, and CSO: expertise in measurement and tool development. This iterative review process helped us to refine the items to improve item quality (e. g., clearer, more concise, mutually exclusive, operational). Three to six items per category were then selected to be included in the questionnaire, using a Likert scale anchored from strongly disagree (1) to strongly agree (6), and included Not Applicable (N/A) in the response options. Selected items were most aligned with our student-centred definitions of each category of validity evidence. We pilot tested the items amongst ourselves to compare how we would answer and discussed what our answers meant. Two items requiring reverse coding were included in the questionnaire (Item 17 and Item 21).

Step IV: We extended the pool of reviewers to close collaborators for an additional review of the questionnaire. More specifically, we requested formal and informal feedback about item clarity and pertinence in relation to our student-relevant validity evidence. In addition, the questionnaire was pilot-tested with research trainees and professionals affiliated with the supervisory team (MY and CSO).

The final version of the questionnaire was web-based and hosted on FluidSurveys. The final questionnaire contained a total of 32 items (22 Likert-type (Tab. 1)), and 10 open-ended questions including questions on: the consequences of failing the assessment, what factors influence performance, study habits and tools used in preparation, the objectives of the examination, comparison of the current exam to other exams in the program, and any additional comments.

### Context

The context of the study was a newly implemented assessment—the Reflection and Evaluation (R&E) Cumulative Multiple-Choice Exam administered in December 2014 to first- and second-year undergraduate medical students. This particular assessment was the focus of this study because it has features that are seen across many assessment tools (cumulative, low stakes, case-based multiple choice question exam). This assessment was designed to encourage integration of knowledge across the curriculum in clinical decision making in a low-stakes manner. Furthermore, this tool was a newly implemented assessment in the curriculum for the class of 2017 and 2018, allowing us the unique opportunity to assess student perceptions of the value of this assessment. More specifically, within the pre-clerkship component of the undergraduate medical program, there are four Reflection and Evaluation Cumulative Exams (R and E exams). Students receive their percentage scores on the examination as their only feedback. They are invited to an exam review session where each question is discussed, and students have the opportunity to review individual examination items and their responses to each question. For this study, we targeted data collected for first and second year students, post exam completion and post grade release. Data collection time points represented Year 1 students’ first experience with the R and E exam and Year 2 students’ penultimate experience with R and E exams. This study, and associated questionnaire, was not intended to provide an in-depth evaluation, nor to determine the quality of the exam itself. Rather, this study, and associated questionnaire, was intended as an exploration of whether students could provide insight regarding how they interpret and make meaning of their assessment scores, how they perceive the role and the quality of assessment through the lens of a validity framework, given their experience with a newly implemented assessment.

### Procedure

All components of this study were approved by the McGill University Institutional Research Ethics Board (A09-E56-11B). Participants in each cohort received an email from their course administrator inviting them to participate in the web-based questionnaire. Prior to beginning, participants were asked to review a consent form and entering the study platform assumed consent. Participants could complete the questionnaire following their examination, following receipt of their scores, or at both time points. Participants were then asked to provide consent to release of their examination scores for the purpose of research. Participants who declined could still complete the questionnaire and were included in analysis.

Following consent, participants completed the questionnaire and volunteered their email address to enter the draw for gift cards. Winners were determined by a random number generator and contacted via email. Participants could exit the online survey at any time they wished, but were unable to complete the survey from their exit point at a later date due to limitations imposed to protect the confidentiality of participant responses. These same limitations prevented us from sending reminders to complete the survey.

### Analysis

#### *Data treatment*:

Two items in the questionnaire were reverse coded, and re-coded appropriately prior to analysis (Tab. 1). Only completed questionnaires were included in the analysis.

#### *Descriptive analysis:*

Descriptive analyses were conducted on questionnaire responses, focused on evidence of validity subscores, and a classical test theory-based item analysis was conducted. Cronbach’s alpha and discrimination coefficients were calculated to inform our interpretation and use of the data. For each category of validity evidence, subscores were generated by taking the average of all responded items within that subscale (i. e. the average of all items a participant provided a response for within that evidence of validity). Factor analysis was not possible due to our limited sample size [30], but subscores were deemed reasonably reliable. Analysis progressed with mean scores per evidence of validity as the primary data of interest.

#### *Student perceptions across types of validity evidence:*

An omnibus repeated measures ANOVA was conducted to investigate the appropriateness of collapsing questionnaire responses across examination cohorts (Year 1 and Year 2), or data collection time point (post examination but prior to score release, or post score release) with the repeated measure of interest being validity evidence subscore. Post-hoc paired t‑tests were conducted to deconstruct main effects.

#### *Relation of questionnaire responses to exam scores:*

To explore whether student perceptions of the value of the examination were related to how well the student performed on the exam, as has been suggested in previous work [21–23], we conducted a correlational analysis investigating the relationship between examination score and responses on the questionnaire. For those participants who consented to release their examination scores, validity evidence subscores were correlated with examination performance.

#### *Analysis of open-ended responses:*

Data analysis was approached as a qualitative description [31], with inductive identification of themes. Coding of the data was done by one co-author (MY) with theme and sub-theme definitions presented iteratively to the research team for refinement throughout analysis until consensus was reached. The resulting themes were intended to be used complementarily to the quantitative data [32] to inform our understanding of the appropriateness of pursuing examinee perception of score use and interpretation in the context of the educational value of assessment.

## Results

### Participants

A total of 104 surveys were completed by 29 Year 1 and 48 Year 2 students. Overall participation rate was 20.5% (77/376). Participation rate amongst Year 1 students was 15.2% (29/191). Participation rate amongst Year 2 students was 25.9% (48/185). A total of 55 students completed surveys before the exam scores were released (14.6%; 55/376) and 49 after the exam scores were released (13.03%; 49/376). Twenty-seven (7.2%) of the students completed surveys both pre- and post-exam scores; overall repeated surveys accounted for 26.0% (27/104) of the total completed surveys.

### Descriptive analysis

Student overall perception of the examination yielded a favourable response with the following overall ratings for each type of validity evidence (Tab. 2): Content (mean = 4.6), Response Process (mean = 4.8), Internal Structure (mean = 4.2), Relationship to Other Variables (mean = 4.0), and Consequential (mean = 4.4).

**Table 2:** Mean ratings per evidence of validity across year, rank ordered from highest mean rating to lowest

Evidence of validity	Mean (SD) ratings for year 1 students	Mean (SD) ratings for year 2 students
*Response process*	4.8 (0.7)^abc^	4.9 (0.8)^a^
*Content*	4.2 (0.9)	4.8 (0.8)^b^
*Consequential*	4.1 (0.9)^a^	4.6 (0.7)^c^
*Internal structure*	4.0 (0.9)^b^	4.3 (0.9)^ab^
*Relation to other variables*	3.7 (1.1)^c^	4.1 (0.9)^abc^

*Same letters (a, b, c) indicate that means are significantly different from each other* (*p*’s < 0.05)

Cronbach’s alpha ranged from 0.54 to 0.84 for the different subscales scales (Content = 0.84; Response Process = 0.54; Structure = 0.77; Relation with Other Variables = 0.73; Consequences = 0.71). Discrimination coefficients ranged from 0.07 to 0.74 and only two items were under the 0.20 threshold set by Ebel and Frisbie [33] as the standard (items 6—specifically targeting exam invigilation, and 23—specifically targeting remediation approaches; see Tab. 1 for specific items). These results support the use of subscores in the subsequent analysis.

### Student perceptions across types of validity evidence

Preliminary analysis suggested that the responses provided by students who completed questionnaires at both time points did not differ significantly across time (*F*(4, 200) = 1.2, *p* = 0.6). Preliminary analysis also suggested that there was no significant difference between questionnaire responses collected prior to, and following, the release of assessment scores (*F*(1, 98)  = 2.6, *p* = 0.1). Therefore, all data points were included regardless of when the questionnaire was completed. Analysis indicated a significant difference across year (Year 1 mean (STD error) = 4.2 (0.1), Year 2 mean (STD error) = 4.5 (0.1); *F*(1, 100) = 7.4, *p* < 0.01), therefore Year was maintained as a factor in subsequent analyses.

**Fig. 1:**
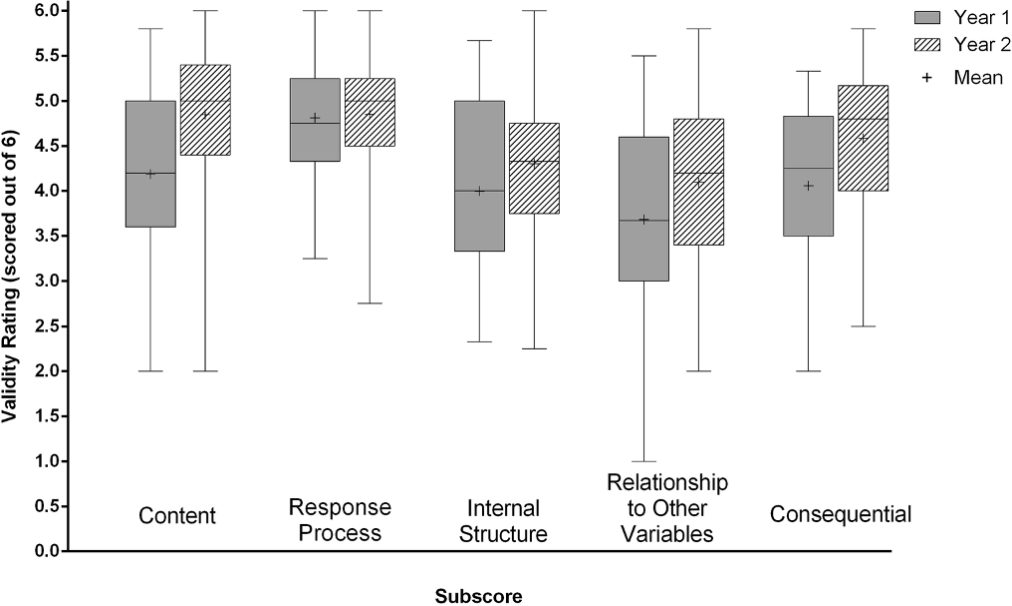
Mean ratings for each validity evidence type, by level of student. Mean is indicated by +, *error bars* represent range

Students responses were significantly different across validity evidence subscore (*F*(4, 392) = 38.3, *p* <0.001), and an interaction was identified between validity subscore and participant year (*F*(4, 392) = 4.3, *p* <0.005). Participants in Year 2 had significantly higher questionnaire scores than Year 1 participants (mean year 2(SD) = 4.5 (0.6), mean year 1(SD) = 4.2(0.8), *F*(1, 98) = 7.9, *p* < 0.01). Mean scores, displayed by participant year can be found in Fig. 1.

Post-hoc paired comparisons revealed ratings were highest for evidence of Response Process and were significantly higher than Consequential, Internal Structure, and Relationship to Other Variables (all *p*’s < 0.005; Tab. 2). Relationship to Other Variables had the lowest mean rating and was significantly lower than Response Process, Content, Internal Structure and Consequential validity (all *p*’s < 0.005; Tab. 2).

### Relationship between survey responses and final grades

A total of 86 participants consented to release their scores and were included in this analysis. Participant examination scores correlated significantly with subscores for Relationship to Other Variables (*r* = 0.34, *p* < 0.005), and Response Process (*r* = 0.24, *p* <0.05), but not with Content (*r* = 0.11, *p* =0.31), Internal Structure (*r* = 0.18, *p* =0.11) nor Consequential (*r* = 0.21, *p* 0.06).

### Student perceptions of assessment

The opportunity to provide ‘other comments’ at the end of the survey proved a rich source of data. Three main themes were identified in the data: 1) the personal effects of the examination on the students, 2) the perceived role of the examination for the institution, and 3) the disappointment in un-met expectations.

#### 1. The personal effects of the examination on the students

For the most part, students report very positive views of the examination, and speak of how well the examination is meeting the educational role of assessment—that the exam is encouraging them to stay ‘up to date’ in their knowledge, integrating various concepts across components of the curriculum, providing feedback on areas of success or areas to improve, supporting confidence building, and an opportunity to apply knowledge outside of content-specific examinations.

*We are given this golden opportunity to stay on top of the knowledge we will be using for the rest of our careers *Participant 73

*[the exam] has contributed to both my learning and my confidence in the development of my skills *Participant 56

*It’s not about the actual exam score, but more that I want to feel as though I have mastered the important concepts *Participant 65

#### 2. The perceive role of the examination for the institution

Students also comment on the apparent role of this examination from the institutional perspective—that it functions as a ‘checkpoint’ for continuance in the curriculum, that it monitors student trajectories of performance, and provides an opportunity for students (and programs) to identify gaps.

*[the exam can] assess our basic understanding of the material into a general comprehension of various clinical presentations that may be similar between various organ systems but different in their pathophysiology and subsequent management *Participant 5

*[the exam can] assure the faculty that all students are following an expected knowledge trajectory *Participant 56

*to allow students to stay up to date on all the material old and new, to put the material into a clinical context, to test that students have an adequate clinical knowledge to move forward in the curriculum *Participant 60

#### 3. The disappointments in un-met expectations

It appears as though the examination was viewed negatively when students perceived that the exam itself was not meeting their expectations as reflected in their description of the roles or goals of the examination.

If students felt that the examination was not meeting its educational role, not supporting learning, and not providing a ‘check point’ for the clinical applicability of their knowledge then they reported dissatisfaction with the exam as a whole. Two sets of comments specifically mentioned the repetition of content across exams—a practice that was purposefully adopted for the examination and was intended to help students continue to improve their knowledge and performance. These participants felt that repetition of content ran counter to the goals of the exam itself—if one repeats content, you are testing specific item memory, and therefore failing the goal of assessing the ability of a student to apply a knowledge base to a clinical scenario.

*(repeated questions) shows I remember the question, but not that my understanding of the material has really increased …. I feel this last exam grade only reflects my ability to recall questions and answers and I am disappointed that I can’t reflect on my true progress *Participant 67

*what I find unacceptable is students having copies of the exam questions …. What will these students do in a clinical setting? More importantly, what will happen to the patients?* Participant 62

Students reported that they perceived that the examination may have been too easy—therefore it was not functioning as a means to identify gaps in knowledge. This is turn was perceived as a missed opportunity for further knowledge acquisition.

*..should be more extensive with harder questions *Participant 49

*while I feel it is easier to keep it this way, I think that having a short answer exam would be much more useful clinically. In the hospital, there will never be only 4 or 5 possible answers to choose from *Participant 79

## Discussion

The goal of this study was to explore whether students could be considered to be valuable stakeholders when conducting a validation of an assessment. Specifically, we conceptualized students as having a voice beyond a simplistic perception of acceptability—that students, as receivers of assessment scores, are responsible for interpreting those scores in the context of evaluating their own performance and as a tool to further their own learning. This consideration of students as stakeholders, and as score interpreters, underpinned the development of a questionnaire aimed at exploring students’ perceived value of an assessment using a student-relevant validity framework. We based the development of our framework on a modern validity theory [24]—and relying on previous work [27], we expanded definitions of the different categories of evidences of validity as defined by Cook et al. [27] to be relevant from an examinee perspective, and developed specific questionnaire items for students to rate when considering a newly implemented integrative assessment.

Participants’ critical comments suggest that the students were engaging with the assessment in a sophisticated manner—including having expectations of the role of this assessment and expressed displeasure when they felt those educational roles were not being filled. These comments suggest that students can engage in judging an examination beyond simple ‘acceptability’. For example, participants had clear expectations of what the assessment ‘should do’ in order to support their learning, and were disappointed when they perceived that these goals were not met. Participants expressed disappointment with an exam that was not (to their view) difficult enough to identify knowledge gaps, or used repeated content and therefore was not testing the application of knowledge that would be required in a clinical setting. This remarkable engagement with, and critical consideration for, the educational goal of assessment emphasizes the potential value of conceptualizing students as meaningful contributors to a validation study if the assessment is intended to have an educational impact. Furthermore, this critical student engagement also provides support for a core tenant of this work—that students are able to provide a well-considered opinion regarding the perceived value and validity of an assessment, given that assessment scores are interpreted in order to support future learning.

Participant ratings differed across categories of validity evidence and ratings were higher on Content, Response Process, Consequential, and Internal Structure while they were lower for Relationship to Other Variables. In our student-relevant conceptualization of validity, Relationship to other variables validity evidence addresses how well the participants felt the exam aligned with future clinical scenarios, other assessments, and future performance. Although the perceived validity of the assessment was favourable, the significantly lower scores for Relationship to Other Variables suggests that students did not necessarily feel that performance on this cumulative examination reflected their future ability to perform in clinical settings, nor related strongly to their performance on other forms of assessment. This was further supported by comments in the open-ended questionnaire items. It seems, at least for these cohorts and this exam, that the appropriateness of an exam includes consideration for how exam performance reflects what is expected of examinees in their future practice. In other words, the value of assessment for learning that will support future clinical practice, may prove to be one of the more dominant characteristics considered by students when judging the validity of an assessment and the educational value of the scores generated by that assessment. Further, if students do not feel that performance on an exam reflects performance in future clinical settings, it may be unlikely that assessment-generated feedback would be integrated to improve later performance. This potential dismissal of assessment-generated feedback was also reflected in the relationship between actual exam performance and perceptions of the assessment’s value. A significant correlation was found between exam score and subscore for Response Process and Relationship to Other Variables—indicating that those who performed more strongly on the exam felt that the exam was more likely to relate to later clinical performance, and more likely to reflect ‘true’ performance than those who received lower examination scores. These lower ratings generated by those who performed less well on the exam suggest that performance and perceptions of an examination are linked, which aligns with previous work [34–36]. Therefore, participants may feel that failing this exam (receiving a low score as feedback) does not necessarily translate into poor clinical skills, knowledge, or judgement, and so may undervalue, or ignore, potential performance-improving feedback derived from this assessment. Further, participants with more experience with this type of examination (the Year 2 participants) did rate the examination significantly higher than those for whom it was their first experience (Year 1 participants).

We often discuss the evidence supporting the validity of an assessment and the roles we expect from assessment, however we may be too rarely attending to assessment as a component of a larger educational process—a process that exists in order to ‘gate-keep’ but also to shape and support learning [9]. Here, we have argued for, and piloted work to contextualize validity evidence within a student-relevant perspective. Assessment can support learning [10, 12] through steering and fostering the learning of an individual student by providing students with performance-based feedback [36–38]. If we contextualize assessment within a feedback framework, it is not only the choice of tool that allows assessment to drive further learning, but the acceptance of the tool, and acceptance of the scores generated by the tool, by those being assessed. The opinion that an examinee holds in regards to the perceived fairness, credibility, or validity of an assessment tool will influence how likely a trainee is to accepting and reflecting on the feedback provided by the assessment to allow for growth [13]. Watling and colleagues demonstrated that the perception trainees have of their assessment process can alter the extent that feedback contributes to learning, even when the assessment tool is based on sound assessment measures [39, 40]. Similarly, when an evaluation process is perceived to be unsatisfactory by those being evaluated, its capacity to foster learning becomes questionable [41]. When a student perceives an assessment process as unfair, they may view an unfavourable outcome negatively and not accept or integrate the feedback provided [23, 34–36], which may hamper future development.

This study has limitations. The first is that the current study had a small sample size; and a sample that may not be representative of the entire medical class. Web-based surveys tend to have a low response rate [42], particularly when no reminders are sent and as such a 20% response rate is within a reasonable range. Further, we do not wish to claim that the findings reported here are generalizable; instead, the purpose of this study was to explore considering students as interpreters of assessment data, therefore opening the possibility of applying validity frameworks (e. g. [24]) to understand student views of assessment quality and educational value. A further limitation is that this study had a single site; however, given the nature of the newly implemented examination (it only exists at a single site), a multi-institution study was not feasible, nor possible. Future work could examine the transferability of the student-relevant validity framework, adapted as needed to a particular assessment context in other settings. Finally, the work reported here was not intended as a full validation study, but rather as an exploration of the notion of students as score interpreters within the framework of assessment *for *learning. As such, we make no claims regarding the validity of the assessment used within this study, nor the ‘accuracy’ of generated scores. Instead, we simply suggest that evidence of validity can be adapted beyond exam administrators as score interpreters, and explore the notion that a validity framework may be applicable to students-as-score-interpreters in the context of the educational role of assessment.

## Conclusions

In this study, we conceptualized students as valuable stakeholders in assessment. Specifically, we considered the roles of students as interpreters of assessment scores—students are the receivers and final ‘actors’ in the assessment process. The recognition of students as having agency within the assessment process may help to contextualize undesirable aspects of student behaviour [15]—cramming to pass the exam, selective studying, and dismissing negative feedback as invalid. The perception that an examination lacks credibility, or value, and the presence of undesirable behaviours on the part of an examinee, may undermine the institutional missions of assessment and decrease the positive impact of sound assessment. Work reported here is a shift from more classic approaches to validity where the ‘interpreters of scores’ are those who administer and monitor assessments, to acknowledging the potential impact of how students interpret and contextualize their assessment scores in order to benefit (or not) later learning. In order to maximize the educational benefits of assessment tools, we must be mindful of unintended negative consequences of assessments, including examinee perceptions of the validity and credibility of assessment.
